# The Ed. Ef Cin. Med. Jour. Remarks

**Published:** 1889-01

**Authors:** 


THE EDITOR OF THE CINCINNATI MEDICAL JOURNAL SENSIBLY REMARKS:—There is great injustice done to a large portion of the medical profession in calling them by any limiting name calculated to place them in a narrow "school," so-called, of medicine. No intelligent, well educated physician should consent to be called an allopath, or homeopath, or hydropath, because these appellations mean, if they mean anything, that the bearer practices in accordance with the narrow tenets of the particular "school."

As a matter of fact the educated physician studies medicine in the broad sense of the term and accepts truth wherever it may be found. Physician, unlimited by any narrowing adjective, is his only proper title; and medicine and surgery in their entirety is the field of his practice.

